# Short-term fasting and fasting mimicking diets combined with chemotherapy: a narrative review

**DOI:** 10.1177/17588359231161418

**Published:** 2023-03-22

**Authors:** Joachim Kikomeko, Tim Schutte, Merel J.M. van Velzen, Rianne Seefat, Hanneke W.M. van Laarhoven

**Affiliations:** Department of Oncology, Amsterdam UMC location Vrije Universiteit Amsterdam, de Boelelaan, Amsterdam, 1117, the Netherlands; Cancer Center Amsterdam, Cancer Treatment and Quality of Life, Amsterdam, the Netherlands; Department of Oncology, Amsterdam UMC location Vrije Universiteit Amsterdam, Amsterdam, the Netherlands; Cancer Center Amsterdam, Cancer Treatment and Quality of Life, Amsterdam, the Netherlands; Department of Oncology, Amsterdam UMC location University of Amsterdam, Amsterdam, the Netherlands; Cancer Center Amsterdam, Cancer Treatment and Quality of Life, Amsterdam, the Netherlands; Division of Molecular Pathology, The Netherlands Cancer Institute, Amsterdam, The Netherlands; Department of Oncology, Amsterdam UMC location University of Amsterdam, Amsterdam, the Netherlands; Cancer Center Amsterdam, Cancer Treatment and Quality of Life, Amsterdam, the Netherlands

**Keywords:** chemotherapy, fasting mimicking diets, short-term fasting

## Abstract

Many patients with cancer search for and use alternative and complementary treatments, aiming to improve the effectiveness of their anticancer treatment and a reduction in treatment-associated side effects. Short-term fasting (STF) and fasting mimicking diets (FMDs) are among the most commonly used dietary interventions. In recent years, different trials have reported the promising results of dietary interventions in combination with chemotherapy, in terms of slowing down tumor growth and reduction in chemotherapy-related side effects. In this narrative review, we identify and describe the current evidence about feasibility and effects of STF and FMDs in cancer patients receiving chemotherapy. The studies that examined the effects of STF when combined with chemotherapy suggest potential benefits regarding reduction in side effects and improved quality of life. We also conclude with a list of well-designed studies that are still recruiting patients, examining the long-term effects of STF.

## Introduction

Many patients with cancer search for and use alternative or complementary treatments to their regular prescribed therapies.^1,2^ Examples of these alternative and complementary treatments not only include herbal medicines and remedies and homeopathy, but also nutritional interventions such as vitamins/minerals, medicinal teas, or specific diets.^2^ Patients use alternative and complementary treatments both with the aim to improve the effectiveness of treatment (by boosting their immune system) and to reduce treatment-associated side effects.^1^ Although a wide range of diets exist, short-term fasting (STF) and fasting mimicking diets (FMDs) are now among the most commonly used dietary interventions; in recent years, different trials have reported the interesting results of dietary interventions in oncology.^3,4^

To support and inform clinicians, we provide a narrative literature review on FMDs complementary to regular therapy for patients with cancer from a clinical perspective.

## What are intermittent fasting and FMDs?

Intermittent fasting refers to episodic periods of little to no calorie consumption. It encompasses a variety of programs that manipulate the timing of eating occasions by utilizing STF. Different patterns include fasting every-other-day, complete 24-h fasting, or fasting on 1 or 2 nonconsecutive days per week. Many fasting programs advise no or limited caloric intake (⩽500 kcal/day) during the fasting period^5^ combined with an unlimited number of calorie-free beverages (i.e. water or coffee). The FMDs are specific meal plans formulated to simulate the fasting state while providing essential nutrients and calories. They are designed to attain fasting-like effects while minimizing the burden of fasting. Specific diets include a variety of plant-based foods designed to satisfy the taste buds. Unlike fasting, FMDs also provide the needed micronutrients such as vitamins and minerals.^6^

## What pathophysiology may be influenced by fasting and FMDs?

Although the exact mechanism is not well understood, weight control by exercise and dietary calorie restriction has been associated with reduced cancer risk.^7^ Cancer cells have a distinct metabolism, with predominant anaerobic use of glucose, which is also known as the Warburg effect.^8^ Therefore, fasting may have different effects on the susceptibility of chemotherapy between healthy cells and cancer cells which is called differential stress resistance.^9–11^ The theory behind STF and FMDs is that these dietary interventions protect healthy cells against stressors, such as chemotherapy while making cancer cells more vulnerable to chemotherapy and other therapies.^12^ Healthy cells can switch toward a maintenance/repair state when nutrients are scarce/absent (such as during fasting), as opposed to cancer cells wherein oncogenes prevent the activation of such stress resistance.^13^ Fasting causes declining plasma levels of insulin-like growth factor-1 (IGF-1),^14^ insulin, and glucose, all vital in preventing apoptosis, promoting growth, accelerating aging, thus leading to cancer.^14^ However, these effects are only induced if fasting persists for a period of at least 48 h.^10,12,15,16^ A molecular explanation for this mechanism is that insulin and IGF-1 signal immunosuppression *via* the STAT3 transcription factor.^17^ As fasting suppresses levels of circulating insulin and IGFs, fasting could (theoretically) lead to less immunosuppression and thus immune activation, as a positive antitumor effect. Besides immunosuppression, STAT3 and the JAK-STAT3 pathways have pleiotropic effects both as a potent tumor promoter and as a tumor suppressor factor associated with cell growth, apoptosis, angiogenesis, invasion, and metastasis.^18^

Plausible molecular pathways have been described to be influenced by fasting and to have effects on cancer biology. The effect of calorie restriction on chemotherapy toxicity was investigated *in vitro* in normal and cancer cells.^19^ In normal cells, serum starvation activates AMPK, which stabilizes p53, resulting in proliferation arrest. During the proliferation, arrest cells are relatively protected from DNA damaging chemotherapy (e.g. CDDP).^19^ In cancer cells, serum starvation additionally activates the ATM/Chk2/p53 stress response signaling pathway.^19^ This hyperactivation of the ATM/Chk2/p53 pathway results in overloading stress which is thought to exhaust cellular stress response and sensitizes cancer cells to DNA damaging chemotherapy.^19^

*In vivo*, calorie restriction in mice led to decreased activity of RAS-mitogen-activated protein kinase (MAPK) and phosphatidylinositol-3-kinase (PI3K)-AKT pathways.^20^ These MAPK and the PI3K/AKT signaling pathways play a very important role in cell survival and proliferation with a potential role in cancer development, differentiation, proliferation, progression, and upregulation of angiogenic cytokines.^21–24^ The observed inhibitory effect on RAS-MAPK and PI3K/AKT pathways provides a possible link between the cancer prevention by the use of calorie restriction. These preclinical findings related to molecular signaling indicate that calorie restriction may have a potential to enhance the therapeutic effect of DNA damaging chemotherapy.

## What is the preclinical evidence for FMDs?

In preclinical studies in mice, FMDs prevented age-related diseases, demonstrated enhanced inflammation, metabolic profile, and increased the life span of the mice.^6^ Moreover, cycles of starvation were as effective as chemotherapeutic agents in delaying progression of different tumor models and increased the effectiveness of chemotherapy in melanoma, glioma, and breast cancer cells.^13^ In mouse models of neuroblastoma, fasting cycles plus cytotoxic drugs, but not either treatment alone, resulted in long-term cancer-free survival.^13^ In mouse models with hormone-receptor-positive breast cancer treated with hormone therapy, FMD also has positive effects, inducing a long-lasting tumor regression and preventing tamoxifen-induced endometrial hyperplasia.^25^ In another breast cancer mouse model (metastatic triple-negative breast cancer of mice injected with 4T1-luc cells), cycles of FMD significantly slowed down tumor growth, reduced tumor size, and caused an increased expression of intratumoral Caspase3, suggesting activation of apoptosis.^26^ This study also showed less disseminated metastasis with predominant lung metastasis in FMD mice, *versus* more disseminated, multiple organ and lymph node metastasis in normal diet mice. Next to significant growth delay, even complete remissions were reported in a part of the mesothelioma xenografts (60%) and lung carcinoma xenografts (40%) following fasting.^19^ Besides effectiveness, mice models also showed fasting protected from leukopenia, heart, kidney, and liver damage when fasted for 24 h before and 24 h after oxaliplatin or doxorubicin administration.^27^ Altogether, preclinical studies have demonstrated the promising effects of fasting and FMDs on chemotherapy effectiveness and protection regarding side effects.

## What are the immunomodulatory effects of fasting and FMD

Fasting and FMDs have been demonstrated to modulate the immune response by various mechanisms. One mechanism demonstrated in a murine breast cancer and melanoma model shows that FMDs enhance the accumulation of tumor-infiltrating lymphocytes and T-cell-mediated tumor cytotoxicity.^28^ Moreover, STF diminishes the levels of circulating IGF-1 to sensitize tumors to programmed cell death protein 1 blockade in preclinical models of non-small-cell lung cancer,^29^ increasing cancer immunogenicity, and boosting antitumor CD8 T-cell responses.^4,28^ In low-immunogenic triple-negative breast cancer models, two cycles of a 4-day FMD were combined with either anti-programmed death-ligand 1 (PD-L1) or a combination of anti-OX40 (costimulatory receptor) and anti-PD-L1. FMD switched the metabolism from glycolysis to oxidative phosphorylation reshaping the tumor micro-environment and enhancing the efficacy of immunotherapy in TNBC tumor types.^30^ The same study also showed an FMD-induced reduction in immune-related adverse events (AEs) by preventing hyperactivation of the immune response. This is a promising nutritional intervention than could be applied to sensitize low-immunogenic tumors to immunotherapy.

## What could be expected by STF and FMDs from a clinical pharmacology perspective?

STF and FMDs introduce relevant pharmacokinetic interactions regarding absorption and metabolism. The absorption is especially relevant for oral formulated oncolytic drugs such as capecitabine [but also for S1, and beyond chemotherapy for oral (targeted) therapies, including tyrosine kinase inhibitors (TKIs)]. For oral chemotherapy, for instance, the rate (*t*_max_) and amount [*C*_max_ and area under the curve (AUC)] of capecitabine are much higher when taken in fasted conditions.^31^ The solubility of, for instance, TKIs is largely dependent on the intragastric acidity (pH Level). Food in the stomach can buffer acidity, thereby increasing intragastric pH. This postprandial intragastric increase in pH can reduce the solubility and absorption of some of these drugs.^32^ For other, for example, lipophilic drugs, concomitant high-fat meals can significantly increase plasma concentrations,^33^ exemplified by lapatinib’s AUC is 425% (after ingestion with a high-fat meal compared to the fasting state.^33^) These observed differences in bioavailability and absolute variability warrant individual recommendation regarding food-fasting and drug interactions in small molecules for which an overview is provided by Veerman *et al*.^34^ Even within one drug class, differences exist. As an example, combining fasting and administration of the oral CDK4/6 inhibitor palbociclib resulted in lower levels and less predictable pharmacokinetic variability. Therefore, the recommendation is that palbociclib should be administered with food. For the other CDK4/6 inhibitors ribociclib and abemaciclib, effects of fasting on drug exposure are insignificant.^35–37^

Regarding altered metabolism, fasting has been shown to influence systemic drug metabolism by both inducing certain cytochrome P450 (CYP) and UGT enzymes (CYP1A2, CYP2D6 CYP3A4, UGT1A4, and UGT2B4/2B7) and reducing enzyme activity of others (2C9).^38^ Although these effects appear to be limited (12–20%), for cytotoxic agents with in general small therapeutic ranges, these fasting-induced changes could be clinically relevant. In one of the included studies, therapeutic drug monitoring was reported; in this study, irinotecan plasma levels were measured and modeled following protein and calorie restriction. The exposure to irinotecan and its active metabolite SN-38, modeled as AUC from zero to 24 h (AUC 0–24 h), was 7.1% and 50.3% higher after protein and calorie restriction *versus* a normal diet, without significant increase in grade ⩾ 3 toxicity.^39^

## Are STF and FMD during chemotherapy safe and feasible?

In six relatively small studies of human subjects (*n* = 10–131) receiving different tumor-specific chemotherapy regimens, STF from 48 to 140 h prior to and 5–56 h following chemotherapy was well tolerated, safe, and feasible,^40–43^ while reducing its toxicity.^11,41,42,44,45^ For an overview of the studies, see Table 1.

**Table 1.** Available human studies investigating the effect of short time Fasting when combined with chemotherapy.

Study	Treatment population	Intervention	Design	Primary endpoint	Results
Safdie *et al.* (2009)^42^	Ten (stage III–IV) cancer patients receiving chemotherapy, 7 females and 3 males with a median age of 61 years, BC (4), prostate cancer (2), ovarian (1), uterine (1), non-small-cell carcinoma of the lung (1), and esophageal adenocarcinoma (1)	Chemotherapy + STF 48–140 h prior and 5–56 h after chemotherapy	Case series	Safety and feasibility (evaluated by blood biomarkers of white blood cell count, absolute neutrophil count, hemoglobin, platelets, creatinine and ALT, total bilirubin, nausea, vomiting, and weight changes).	Significant reduction in fatigue and weakness (*p* < 0.001 and *p* < 0.00193, respectively), whereas vomiting and diarrhea were virtually absent in combination with fasting None of these patients, who received an average of four cycles of various chemotherapy drugs in combination with fasting, reported significant side effects caused by the fasting itself other than hunger and lightheadedness
Badar *et al.* (2014)^41^	11 cancer patients receiving chemotherapy, 9 females and 2 males with a median age of 44 years (range 27–51 years), BC (3), non-Hodgkin lymphoma (2), acute myeloid leukemia (1), nasopharynx (1), ovarian (1), and colon cancer (1)	Patients receive their chemotherapy 20 min after sunset, and then allowed to continue their routine fasting schedule for the rest of the month (i.e. fast daily from dawn to sunset and eat and drink from sunset to dawn) After a ‘wash out’ period of at least 2 weeks after end of Ramadan, patients receive the same chemotherapy while not fasting	One-group non-randomized, cross-over, open-label clinical trial	Safety and feasibility Evaluated for symptoms and blood biomarkers pre- and post-chemotherapy while fasting and non-fasting	No serious complications among patients in fasting group No alarming spikes/changes in blood biomarkers or deterioration of symptoms during fasting compared to non-fasting
de Groot *et al.* (2015)^44^	13 patients with HER2-negative stage II/III BC, with a median age of 51 years (STF) and 52 years (non-STF)	Six cycles chemotherapy (ACT) ± 48 h STF	RCT	Feasibility of STF, effect on tolerance of chemotherapy	STF during chemotherapy was well tolerated and reduced hematologic toxicity of TAC in HER2-negative BC patients. STF may reduce a transient increase in, and/or induce a faster recovery of DNA damage in PBMCs after chemotherapy Mean erythrocyte and thrombocyte counts 7 days post-chemotherapy were significantly higher (*p* = 0.007, 95% CI: 0.106–0.638 and *p* = 0.00007, 95% CI: 38.7–104, respectively) in the STF group compared to the non-STF group
Dorff *et al.* (2016)^45^	20 patients with different malignancies, median age was 61 years, and 85% were women	Platinum-based chemotherapy +24, 48, or 72 h STF Dose-escalation study of fasting in human cancer patients receiving chemotherapy (24–72 h): restriction of calorie intake <200 kcal per 24 h	RCT	Feasibility	There was a non-significant trend toward less grade 3 or 4 neutropenia in the 48- and 72-h cohorts compared to 24-h cohort (*p* = 0.17) Fasting for 72 h around chemotherapy administration is safe and feasible for cancer patients IGF-1 levels decreased by 30%, 33%, and 8% in the 24-, 48-, and 72-h fasting cohorts, respectively, after the first fasting period
Bauersfeld *et al.* (2018)^11^	34 women (BC), 4 patients (ovarian cancer), median age 51 years (range 28–69 years	Different chemotherapy regimens (ACT, epirubicin, methotrexate, fluorouracil) with/without ±60-h STF Fasting = unrestricted amounts of water, herbal tea, 2 × 100 cal vegetable juice and small standardized quantities of light vegetable broth with a maximum total daily energy intake of 350 kcal	Randomized cross-over trial	Feasibility + effects on QoL	STF during chemotherapy is well tolerated and appears to improve QoL and fatigue The mean chemotherapy-induced deterioration of total FACIT-F was 10.4 ± 5.3 for fasted and 27.0 ± 6.3 for non-fasted cycles in group A and 14.1 ± 5.6 for non-fasted and 11.0 ± 5.6 for fasted cycles in group B
Riedinger *et al*. (2020)^40^	20 women with gynecologic malignancies receiving at least 6 planned chemotherapy cycles	Fasting patients maintaining a water-only fast for 24 h before and 24 h following each chemotherapy cycle were compared to non-fasting patients	RCT	Feasibility and effect of STF in patients receiving chemotherapy	Fasting resulted in fewer treatment modifications and improved quality of life scores over the course of treatment No significant difference in mean QoL scores between the fasting and control group 58.38 *versus* 57.10, *p* = 0.7
de Groot *et al.* (2020)^12^	131 patients with HER2-negative stage II/III BC, median age 49 years in FMD and 51 years in regular diet	FMD or regular diet for 3 days prior to and during neoadjuvant chemotherapy FMD is a 4-day plant-based low amino acid substitution diet, calorie content declined from Day 1 (~1200 kcal), to Days 2–4 (~200 kcal)	RCT	Toxicity and disease response (radiologically and pathologically)	No difference in toxicity between both groups, despite the fact that dexamethasone was omitted in the FMD group Radiologically complete or partial response occurs more often in patients using the FMD (OR: 3.168, *p* = 0.039) Pathological response is more likely to occur in patients using the FMD (OR: 4.109, *p* = 0.016)
Zorn *et al.* (2020)^46^	51 patients with gynecologic cancers, median age of 54 years	Patients with a minimum of four cycles fasted for 96 h during half of their chemotherapy cycles and consumed a normocaloric diet during the other chemotherapy cycles Caloric restriction was limited to 25% of each patient’s daily calorie. This was between 400 and 600 kcal/day for most patients + minimum of 2.5 L water	Controlled cross-over trial	Chemotherapy-induced toxicities, fasting-related discomfort, body composition, QoL, laboratory values, and compliance	mSTF was safe and feasible. Significant reduction in total toxicities’ score (−10.36 ± 4.44; 95% CI: −19.22 to −1.50; *p* = 0.023). Less chemotherapy postponements post-mSTF, reflecting improved tolerance of chemotherapy (−0.80 ± 0.37; 95% CI: −1.53 to −0.06; *p* = 0.034)
Ligorio *et al.* (2022)^43^	101 patients with different tumor types with different cancer types, disease stages, and receiving different types of standard antineoplastic treatments. Median age: not reported but 70% <60 years	All patients received a 5-day, plant-based, calorie-restricted (up to 600 kcal on Day 1; up to 300 kcal on Days 2, 3, 4, and 5), low-carbohydrate, low-protein dietary regimen that was repeated every 3/4 weeks, up to a maximum of eight consecutive FMD cycles	Open label, single-arm, prospective clinical trial	Evaluating the safety, feasibility, metabolic, and immunomodulatory effects	After median follow-up of 44.2 months (95% CI: 38.4–50.6), 34 death events occurred in these 69 patients, mOS was 29.9 months (95% CI: 23.6–NR). No events were the result of acute or chronic FMD-related AE. 6 (7.9%) patients were defined as exceptional responders
Valdemarin *et al.* (2021)^47^	90 patients with 18 different types of cancer ( solid or hematologic), low nutritional risk and undergoing active medical treatment, median age not indicated	Patients received periodic FMD cycles. The body weight, handgrip strength, and body composition were monitored throughout the study. Growth factors, adipokines, and cyto/chemokines were assessed. FMD was a 5-day, low-calorie and low-protein diet that supplies approximately 1099 kcal on Day 1 (11% protein, 46% fat, and 43% carbohydrates), approximately 3000 kJ (717 kcal) (9% protein, 44% fat, and 47% carbohydrates) on Days 2–5	Open label, single-arm, phase I/II clinical trial	Evaluating the safety and feasibility of FMD	FMD was largely safe with only mild side effects. The patients’ weight and handgrip remained stable, the phase angle and fat-free mass increased, while the fat mass decreased. FMD reduced the serum c-peptide, IGF-1, IGF-BP3, and leptin levels, while increasing IGF-BP1, and these modifications persisted for weeks beyond the FMD period
Vernieri *et al.* (2022)^4^	101 patients with any malignancy, with the exception of small-cell neuroendocrine tumors, 72% <60 years	The FMD was administered up to a maximum of 8 consecutive cycles in combination with standard adjuvant treatments or therapies for advanced disease. FMD was a 5-day plant-based, low-calorie (600 kcal on Day 1, followed by 300 kcal/day on Days 2–5), low-protein, low-carbohydrate diet	Open label, single-arm, prospective clinical trial	The safety and biological effects of cyclic, 5-day FMD in combination with standard antitumor therapies	Severely calorie-restricted, low-carbohydrate, low-protein, 5-day dietary regimen that mimics fasting is safe and feasible when repeated every 21–28 days in combination with standard antitumor treatments, and it reshapes systemic metabolism and antitumor immunity
de Man *et al.* (2020)^39^	19 patients with liver metastases of solid tumors, median age is 65 years	Included patients were randomized to treatment with irinotecan preceded by 5 days of PCR (~30% caloric and ~70% protein restriction) during the first cycle and a second cycle preceded by a normal diet or vice versa. The pharmacokinetic blood sampling and biopsies of both healthy liver and liver metastases were performed	Randomized, two-armed, pharmacokinetic crossover clinical trial	The difference in geometric means for the active metabolite SN-38 concentration in healthy liver analyzed	No significant differences were seen in irinotecan (+16.8%, *p* = 0.22) and SN-38 (+9.8%, *p* = 0.48) concentrations between PCR and normal diet in healthy liver, as well as in liver metastases (irinotecan: −38.8%, *p* = 0.05 and SN-38: −13.8%, *p* = 0.50). PCR increased irinotecan plasma AUC 0–24 h with 7. 1% (*p* = 0.04) compared with normal diet, whereas the SN-38 plasma AUC 0–24 h increased with 50.3% (*p* < 0.001). Grade ⩾ 3 toxicity was not increased during PCR *versus* normal diet (*p* = 0.69). No difference was seen in neutropenia grade ⩾ 3 (47% *versus* 32% *p* = 0.38), diarrhea grade ⩾ 3 (5% *versus* 21% *p* = 0.25), and febrile neutropenia (5% *versus* 16% *p* = 0.50) during PCR *versus* normal diet

Feasibility was defined as ⩾50% subjects in each cohort consuming ⩽200 kcal per 24 h during the fast period without excess toxicity.

ACT, doxorubicin, cyclophosphamide, docetaxel; AE, adverse event; ALT, alanine transaminase; AUC, area under the curve; BC, breast cancer, CI, confidence interval; FMD, fasting mimicking diet; IGF, insulin-like growth factor; mOS, median overall survival; OR, odds ratio; PCR, protein and calorie restriction; QoL, quality of life; RCT: randomized controlled trial; STF: short-term fasting.

Fasting for 48 h is well tolerated without increasing weight loss, hospital admissions, or chemotherapy dose reduction/delays according to a randomized control trial in women with gynecologic malignancies receiving at least six planned chemotherapy cycles.^40^ Fasting patients maintaining a water-only fast for 24 h before and 24 h following each chemotherapy cycle were compared to non-fasting patients. Treatment-related side effects and quality of life (QoL) were assessed using NCCN-FACT FOSI-18 questionnaire. Ninety percent of the patients received taxane- and platinum-based doublet therapy. Weight loss and unanticipated hospitalizations were similar between treatment groups. There were fewer dose reductions or delays registered in the fasting group and fasting did not result in significant weight loss, even in a patient population at significant risk for malnutrition.^40^

A case series of 10 patients with different malignancies undergoing therapy combined with STF also demonstrated safety and feasibility of STF. (42) All 10 cases fasted for 48–140 h prior to and/or 5–56 h following chemotherapy. None of these patients, who received an average of four cycles of various chemotherapy drugs in combination with fasting, reported significant side effects caused by the fasting itself other than hunger and lightheadedness.^42^

In another clinical study evaluating safety, tolerability, and QoL of combining chemotherapy with STF, better tolerance to chemotherapy was reported^11^. This study included women with breast cancer and ovarian cancer and used an intra-individual randomized cross-over study design to balance for the heterogeneity in disease states and chemotherapy protocols. STF with a period of 60 h was not associated with weight loss and was associated with only minor adverse effects that were rated as not meaningful by the patients and did not interfere with daily activities.

When combined with chemotherapy and standard antineoplastic treatments in cancer patients at low nutritional risk, periodic FMD cycles are feasible.^4,2,25,43^ An overview of these in-human FMD studies is given in Table 1.

## What is the effectiveness of intermittent fasting and FMDs with regard to protection against toxicity and chemotherapy-related side effects?

A number of studies (see Table 1 for overview) have investigated the effectiveness of FMDs with regard to protection against toxicity, chemotherapy-related side effects, and QoL.^11,12,40,42–44^ A randomized control trial was conducted in women with gynecologic malignancies receiving at least six planned chemotherapy cycles combined with STF. Fasting during chemotherapy led to an improvement in patient-reported QoL scores over the course of chemotherapy treatments.^40^ Although this study was not powered to detect further differences in outcomes, there was a trend toward fewer hospitalizations, improvement in hematologic parameters, and fewer dose reductions or delays in treatment for the fasting group.

In a small and heterogeneous group of patients during and after Ramadan, fasting was well tolerated and comparable reduction in side effects compared to non-fasting period was reported.^41^ Eleven cancer patients receiving chemotherapy were recruited for this pilot study. Patients were allowed to continue their routine fasting schedule while receiving chemotherapy. After a ‘wash out’ period of at least 2 weeks after the end of Ramadan, patients received the same chemotherapy while not fasting. All patients were interviewed by phone daily regarding eventual chemotherapy side effects, and a complete blood count as well as renal and liver function monitored once weekly. All patients reported fewer side effects in the chemotherapy period during Ramadan.^41^

In women with breast cancer and ovarian cancer, STF led to a better tolerance to chemotherapy with less compromised QoL (FACIT-measurement system) and reduced fatigue 8 days after chemotherapy.^11^ In this randomized cross-over trial, gynecologic cancer patients with four to six planned chemotherapy cycles were included. In all, 34 patients were randomized to STF in the first half of chemotherapy treatment followed by a normocaloric diet (group A; *n* = 18) or vice versa (group B; *n* = 16). Fasting started 36 h before and ended 24 h after chemotherapy (60-h fasting period). Next to better chemotherapy tolerance, STF was not associated with any serious side effects.^11^ Similar findings were reported in a study of 13 women with HER2-negative breast cancer who were treated with chemotherapy.^44^ Eligible patients with HER2-negative stage II/III breast cancer receiving (neo)-adjuvant chemotherapy (docetaxel/doxorubicin/cyclophosphamide) were randomized to fast 24 h before and after commencing chemotherapy, or to eat according to the guidelines for healthy nutrition. Chemotherapy-induced DNA damage in peripheral blood mononuclear cells (PBMCs) was quantified by the level of γ-H2AX (a sensitive molecular marker of DNA damage and repair) analyzed by flow cytometry.^44^ STF was well tolerated and mean erythrocyte and thrombocyte counts after 7 days of chemotherapy were significantly higher in the STF group compared to the non-STF group. Levels of γ-H2AX were significantly increased 30 min post-chemotherapy in CD45+ CD3− cells in non-STF, but not in STF patients. STF may reduce a transient increase and/or induce a faster recovery of DNA damage in PBMCs after chemotherapy. Non-hematologic toxicity did not differ between the groups.

The DIRECT trial investigated the effect of the addition of a special a low-calorie and protein-restricted diet (Chemolieve) to chemotherapy on side effects and effect of chemotherapy (four courses of AC followed by four courses of docetaxel) in patients with breast cancer.^12^ In all, 131 patients with HER2-negative stage II/III breast cancer were randomized to receive either a FMD or their regular diet for 3 days prior to and during neo-adjuvant chemotherapy. Results showed no difference in toxicity between both groups, despite the fact that dexamethasone was omitted in the FMD group. FMD may alleviate the need for prophylactic treatment with dexamethasone for the prevention of chemotherapy side effects. Results of the secondary outcomes of the DIRECT trial^48^ reported improvement of certain QoL and illness perception domains in patients receiving FMD as an adjunct to neoadjuvant chemotherapy. The incidence of severe toxicity (Common Terminology Criteria for Adverse Event grade ⩾ 3) in patients treated with irinotecan on a protein and calorie restriction seemed higher (53%) compared to a normal diet (42%), although this trend was not statistically different (*p* = 0.69).^39^

In the most recent study by Vernieri *et al*., 10 patients receiving *n* with standard antitumor therapies were assigned a cyclic 5-day FMD regimen. The trial reported good compliance and no increased safety concerns. The trial met its primary endpoint, with an incidence of severe grade 3 or 4 FMD-related AEs of 12.9% (90% confidence interval: 7.8–19.7%), significantly lower than the pre-specified 20% threshold.^4^ A comparable safety profile was observed by Valdemarin *et al*.^47^. In this single-arm, phase I/II clinical trial, 100 patients with solid or hematologic malignance underwent active medical treatment combined with periodic FMD cycle. No grade 3–5 AEs related to FMD were observed.

## What is the effect of STF on the response to chemotherapy?

There is only one randomized controlled study that has evaluated the effects of an FMD on efficacy of chemotherapy in human patients with cancer (DIRECT trial, described in the previous section).^12^. An FMD improved the clinical response to neoadjuvant chemotherapy as compared to a regular diet in HER2-negative early breast cancer patients receiving chemotherapy.^12^ Radiologically complete or partial response occurs more often in patients using the FMD [odds ratio (OR): 3.168, *p* = 0.039] and a pathological response was more likely to occur in patients using the FMD (OR: 4.109, *p* = 0.016). The results of DIRECT trial demonstrated that FMDs could make dexamethasone pre-medication unnecessary in the prevention of chemotherapy-induced nausea and vomiting.^12^ This could be very important because glucocorticoids including dexamethasone are thought to promote breast cancer metastasis.^49^ By avoiding dexamethasone, FMDs could thus potentially strengthen anticancer effects of chemotherapy.

## What is the optimal duration of FMD or STF?

The duration of fasting intervals of STF in the different studies ranged from 48 to 140 h.^11,12,42,44,45^ This is the only study that evaluated different regimens/durations of STF during the investigation of 20 patients with three different fasting periods (24, 48, and 72 h).^45^ In this trial, 16 of the patients were compliant with the fasting regimen (<200 kcal/day) and showed reduced DNA damage in (host)leukocytes after chemotherapy exposure for subjects who fasted 72 h compared to 24 h. For the FMDs, four trials evaluated comparable durations of FMDs of 4 days^12^ (DIRECT trial) and 5 days,^4,43,47^ the included tumor types in these studies differed. The 5-day FMD regimen showed a better adherence, although the observed difference could be explained by the different calorie content and patient characteristics differed (see Table 1 and separate subheading).

## What is the optimal FMD regimen?

Most studies evaluating FMDs all had a bi-phasic regimen of 4–5 days with a medium restriction on Day 1 with further caloric restriction on the following days. Dietary contents of the FMDs vary and range between 1200 and 600 kcal on Day 1 followed by a restriction between 700 and 200 kcal/day. approximately 1099 kcal on Day 1 (11% protein, 46% fat, and 43% carbohydrates), approximately 717 kcal (9% protein, 44% fat, and 47% carbohydrates) on Days 2–5.^47^ The DIRECT study FMD regimen comprised of 1200 kcal on Day 1 and 200 kcal on Days 2–4.^12^ In the most recent publication by Vernieri *et al.*, a 5-day FMD regimen comprising of 600 kcal on Day 1, up to 300 kcal on Days 2–5 was applied. Most FMDs consist of plant-based ingredients and the nutritional contents vary (see Table 1).

Compliance is a major issue regarding FMD use in combination with chemotherapy. It varied between studies.^4,12,47,50^ Unlike the DIRECT trial that reported poor FMD compliance of 20% for all planned FMD cycles,^12^ other recent human oncologic and non-oncologic trials have reported much better compliance of up to 72% (see overview in Table 1).^4,47,50^ These observed differences in compliance could be explained by differences in FMD composition and patient management. Given the heterogeneity and nature of the reported trials, with no head-to-head comparisons, the optimal FMD regimen including the timing, the amount of caloric restriction, and which nutrients should be restricted/preserved is unknown.

## Which patients were included in STF and FMD trials?

The clinical trials described in this article tend to suggest a positive trend in support of the combination in patients receiving cancer treatment for different malignancies. When combined with chemotherapy, STF or FMD could provide treatment-related benefits in patient patients with breast cancer^4,11,12,43,44^ and gynecologic cancers.^11,40,46^ The two most recent trials were less selective as they included patients with >10 different malignancies on different anticancer therapy.^4,47^

## Which clinical trials of FMDs and STF are ongoing within clinical oncology?

As the field of clinical oncology and complementary diets is rapidly evolving, in Table 2, we summarize the registered trials researching FMDs and STF within clinical oncology. We identified nine trials currently registered with different oncologic conditions (lung, breast, ovarian, prostate, and colorectal cancer) ranging from stage II to stage IV. The primary endpoints are pathological response, QoL, optimal fasting duration, and grade 2–4 side effects. The expected number of participants varies per study ranging from 12 to 150 participants with estimated study completion date ranging from 2022 to 2024.

**Table 2.** 

Status	Study title	Conditions	Interventions	Design	Primary endpoint	Estimated enrolment	Actual start date
Recruiting NCT04248998	Calorie Restriction With or Without Metformin in Triple Negative Breast Cancer BREAKFAST trial	Triple-negative breast cancer	FMD with or without Metformin	RCT	Rate of pCRs	90 participants	5 May 2020
Recruiting NCT03162289	Intermittent Fasting Accompanying Chemotherapy in Gynecological Cancers (FIT2)	Breast cancer and ovarian cancer	Fasting: fasting regime of 60–72 h (36–48 h before and 24 h after CT) Vegan [60–72 h vegan diet with sugar restriction (36–48 h before and 24 h after CT)]	RCT	Summarized change in FACT-G score	150 participants	10 May 2017
NCT00936364	Short-Term Fasting: Impact on Toxicity	Malignant solid tumors	STF/modified fast fasting	RCT	Identification of the longest duration of fasting which is safe	70 participants	9 July 2009
Active, not recruiting NCT02710721	Clinical Study on the Efficacy of Fasting and Nutritional Therapy as a Complementary Treatment of Advanced Metastatic Prostate Cancer Undergoing Chemotherapy—an Exploratory Randomized Controlled Trial	Castration-resistant prostate cancer or hormone-sensitive metastatic prostate cancer with high disease burden	A 60-h-modified fasting (36 h before and 24 h after chemotherapy) with a dietary energy supply 350–400 kcal per day (Chemolieve)	RCT	Summarized change in FACT score from baseline to Day 8 after each chemotherapy	60 participants	April 2016
Unknown NCT04027478	Can Fasting Decrease the Side Effects of Chemotherapy	Patients undergoing chemotherapy with Taxol/carboplatin planned for at least six cycles	FMD consisting of 10 kcal/kg/day and including 50% fat, 40% carbohydrates, and no more than 10% protein	RCT	Grade 2–4 nausea when patients are in the FMD sequence *versus* the non-fasting sequence	39 participants	1 September 2019
New NCT05327608	Intermittent Fasting for the Improvement of Outcomes in Patients With Stage I–III Breast Cancer Receiving Chemotherapy Before Surgery	Stage I–III breast cancer receiving chemotherapy before surgery	STF Chemotherapy	Interventional (Clinical Trial) Single Group Assignment	Proportion of patients that can adhere to intermittent fasting	50 participants	1 May 2022
Enrolling by invitation NCT04247464	Short-term Fasting as an Enhancer of Chemotherapy: Pilot Clinical Study on Colorectal Carcinoma Patients	Colorectal carcinoma	Fasting Chemotherapy	RCT	Changes in the CTC-AE 5.0 toxicity table score	100 participants	23 September 2020
Recruiting NCT03700437	Randomized Controlled Pilot Study to Evaluate Fasting-mimicking Diet in Patients Receiving Chemo-immunotherapy for Treatment of Metastatic Non-small-Cell Lung Cancer	Stage IV lung adenocarcinoma treated with carboplatin/pemetrexed and pembrolizumab	Chemolieve® for 3 days prior to and on the day of chemo-immunotherapy during the first four cycles	Interventional (Clinical Trial) Single Group Assignment	Proportion of the patients who can finish the FMD without serious AEs	12 participants	2 November 2018
Unknown NCT03595540	Phase II Clinical Study of a Fasting-Mimicking Diet in Patients Undergoing Oncologic Treatment	Solid or hematologic tumors undergoing active treatment	Prolon by L-Nutra concomitantly with standard antitumor therapies	Interventional (Clinical Trial) Single Group Assignment	Percentage of prescribed diet consumed and intake of any extra food	60 participants	22 November 2017

ClinicalTrials.gov. Available online: Search of: chemotherapy, fasting | Recruiting, Not yet recruiting, Active, not recruiting, Completed, Enrolling by invitation, Unknown status Studies | Cancer | Adult - List Results – ClinicalTrials.gov accessed on 01 June 2022.

FACT-G: Functional Assessment of Cancer Therapy – General (FACT-G) is a 27-item questionnaire designed to measure four domains of HRQoL in cancer patients: Physical, social, emotional, and functional well-being.

AEs, adverse events; CTC-AE, Common Terminology Criteria for Adverse Event; FMD, fasting mimicking diet; pCRs, pathologic complete responses; RCT, randomized controlled trial; STF, short-term fasting.

## Conclusion and future directions

Altogether, STF and FMDs are promising dietary interventions; however, uncertainty persists regarding the safety, feasibility, and effectiveness of fasting on the response to chemotherapy for the general population. Especially STF including water-only fasting can introduce safety issues when sustained for a longer time and can reduced adherence. Based on previous trials, specific knowledge gaps in literature include the optimal fasting schedule (timing, amount of caloric restriction and which nutrients should be restricted). Ideally, a large randomized clinical trial would compare the a standardized FMD and a control arm a normal diet all combined with standard chemotherapy with progression-free survival, overall survival, and side effects as its endpoints. Such a trial would ideally need to include a measure to assess compliance. The observed compliance in recent trials demonstrate high FMD compliance can be reached by dietician support. Realistically, such trials are hard to fund and organize and individualized counseling of patients regarding their wishes for complementary care should remain the standard of care. Healthcare professionals in oncology should therefore be adequately informed of risks and benefits of dietary interventions as complementary cancer treatment.

## Declarations

**Ethics approval and consent to participate:** This article does not contain any studies with humans or animals performed by any of the authors. All studies described in this review were referenced.

**Consent for publication:** All authors consented to publication of the final draft of this article.

**Availability of data and materials:** Data sharing is not applicable to this article as no new data were created or analyzed in this study.
